# Elevated phospholipids and acylcarnitines C4 and C5 in cerebrospinal fluid distinguish viral CNS infections from autoimmune neuroinflammation

**DOI:** 10.1186/s12967-023-04637-y

**Published:** 2023-11-02

**Authors:** Amani Al-Mekhlafi, Fakhar H. Waqas, Maike Krueger, Frank Klawonn, Manas K. Akmatov, Kirsten Müller-Vahl, Corinna Trebst, Thomas Skripuletz, Martin Stangel, Kurt-Wolfram Sühs, Frank Pessler

**Affiliations:** 1 Biostatistics, Helmholtz Centre for Infection Research, Braunschweig, Germany; 2 Research Group Biomarkers for Infectious Diseases, TWINCORE Centre for Experimental and Clinical Infection Research, Feodor-Lynen-Str. 7, 30625 Hannover, Germany; 3 Research Institute of Ambulatory Care, Berlin, Germany; 4 Department of Psychiatry, Social Psychiatry and Psychotherapy, Hannover Medical School, Hannover, Germany; 5 Department of Neurology, Hannover Medical School, Hannover, Germany; 6 Centre for Individualised Infection Medicine, Hannover, Germany; 7 Present Address: Translational Medicine, Novartis Institute for Biomedical Research, Basel, Switzerland

**Keywords:** Autoimmunity, Biomarkers, Cell membrane, Central nervous system, Fatty acid oxidation, Encephalitis, Enterovirus, Glycerophospholipids, Herpes simplex virus, Infection, Isobutyrylcarnitine, Isovalerylcarnitine, Lecithin, Lysophosphatidylcholine, Meningitis, Metabolism, Mitochondria, n-methyl-D-aspartate receptor, Phosphatidylcholine, Phospholipid, Sphingomyelin, Varicella zoster virus

## Abstract

**Background:**

Viral and autoimmune encephalitis may present with similar symptoms, but require different treatments. Thus, there is a need for biomarkers to improve diagnosis and understanding of pathogenesis. We hypothesized that virus-host cell interactions lead to different changes in central nervous system (CNS) metabolism than autoimmune processes and searched for metabolite biomarkers in cerebrospinal fluid (CSF) to distinguish between the two conditions.

**Methods:**

We applied a targeted metabolomic/lipidomic analysis to CSF samples from patients with viral CNS infections (n = 34; due to herpes simplex virus [n = 9], varicella zoster virus [n = 15], enteroviruses [n = 10]), autoimmune neuroinflammation (n = 25; autoimmune anti-NMDA-receptor encephalitis [n = 8], multiple sclerosis [n = 17), and non-inflamed controls (n = 31; Gilles de la Tourette syndrome [n = 20], Bell’s palsy with normal CSF cell count [n = 11]). 85 metabolites passed quality screening and were evaluated as biomarkers. Standard diagnostic CSF parameters were assessed for comparison.

**Results:**

Of the standard CSF parameters, the best biomarkers were: CSF cell count for viral infections vs. controls (area under the ROC curve, AUC = 0.93), Q-albumin for viral infections vs. autoimmune neuroinflammation (AUC = 0.86), and IgG index for autoimmune neuroinflammation vs. controls (AUC = 0.90). Concentrations of 2 metabolites differed significantly (p < 0.05) between autoimmune neuroinflammation and controls, with proline being the best biomarker (AUC = 0.77). In contrast, concentrations of 67 metabolites were significantly higher in viral infections than controls, with SM.C16.0 being the best biomarker (AUC = 0.94). Concentrations of 68 metabolites were significantly higher in viral infections than in autoimmune neuroinflammation, and the 10 most accurate metabolite biomarkers (AUC = 0.89–0.93) were substantially better than Q-albumin (AUC = 0.86). These biomarkers comprised six phosphatidylcholines (AUC = 0.89–0.92), two sphingomyelins (AUC = 0.89, 0.91), and acylcarnitines isobutyrylcarnitine (C4, AUC = 0.92) and isovalerylcarnitine (C5, AUC = 0.93). Elevated C4 and C5 concentrations suggested dysfunctional mitochondrial β-oxidation and correlated only moderately with CSF cell count (Spearman *ρ* = 0.41 and 0.44), indicating that their increase is not primarily driven by inflammation.

**Conclusions:**

Changes in CNS metabolism differ substantially between viral CNS infections and autoimmune neuroinflammation and reveal CSF metabolites as pathophysiologically relevant diagnostic biomarkers for the differentiation between the two conditions. In viral CNS infections, the observed higher concentrations of free phospholipids are consistent with disruption of host cell membranes, whereas the elevated short-chain acylcarnitines likely reflect compromised mitochondrial homeostasis and energy generation.

**Supplementary Information:**

The online version contains supplementary material available at 10.1186/s12967-023-04637-y.

## Introduction

Encephalitis continues to exert a strong disease burden and to present diagnostic challenges. The incidence of encephalitis is about 10–20 per 100,000 in the United States [1], and approximately 7 of 100.000 are hospitalized due to encephalitis [2]. Etiology remains unspecified in about half the cases [3], but 20–50% of cases with known etiology are caused by viruses. Incidence and pathogen spectrum vary significantly depending on geographic region. In the United States and Europe most of the cases can be attributed to herpes simplex virus (HSV), followed by varicella zoster virus (VZV) and enteroviruses. The prognosis depends strongly on timely diagnosis and institution of correct treatments. For example, HSV encephalitis-associated lethality is 70–100% in untreated patients but 20–30% in patients receiving virus specific therapy [4]. Therefore, attempts have been made to improve the detection of viral pathogens. Besides PCR and multiplex assays, metagenomic next-generation sequencing (mNGS) could prove useful, but it appears to be less accurate for the detection of viral (AUC = 0.5–0.7) than bacterial pathogens (AUC = 0.85) [5].

Despite the sequelae evoked by the primary virus infection itself, viral relapses can occur as well as secondary autoimmune processes leading to autoimmune neuroinflammation. In the case of HSV encephalitis, about 25% of all patients experience a relapse either by virus reactivation or, in 15–20% by an autoimmune anti-n-methyl-D-aspartate-receptor (NMDAr) encephalitis [6, 7]. In autoimmune encephalitis the host immune system targets self-antigens expressed in the central nervous system (CNS) [8]. The symptoms vary based on the specific autoantibody involved, yet shared characteristics collectively suggest the likelihood of autoimmune-driven encephalitis [9]. However, symptoms of viral and autoimmune CNS inflammation are similar (e.g. altered mental status, seizures) and the etiology can therefore not be differentiated on the basis of clinical findings.

Advances in diagnostics have enabled us to identify several well-characterized autoantibodies that cause autoimmune encephalitis, but the results are not available in the acute setting, as turnaround time may be up to 1–2 weeks depending on work flows in diagnostic laboratories. Moreover, about 50% of encephalitis patients remain without etiological diagnosis and it is unknown how many of these actually suffer from an autoimmune encephalitis. Considering that viral and autoimmune encephalitis require completely different treatments (antivirals vs. immune suppression/apheresis), there is a clear need for diagnostic biomarkers to distinguish between these two entities as soon as possible after lumbar puncture. Apart from the detection of a viral pathogen or a specific autoantibody, there are several biomarkers that aid to differentiate viral from autoimmune encephalitis. CSF leukocyte count is one of the best-known CSF markers, and together with clinical parameters (subacute/chronic presentation; Charlson comorbidity index < 2 and psychiatric/memory complaints) the absence of inflammatory changes in CSF has been used to generate a risk score for autoimmune encephalitis (area under the ROC curve, AUC, 0.92 [95% CI, 0.87–0.97]) [10]. CSF oligoclonal bands are more common in patients with autoimmune encephalitis than viral encephalitis, yet can be found in non-inflammatory diseases as well [11]. Cytokines such as M-CSF and the B-cell markers CXCL13 and BAFF tend to be elevated in autoantibody-associated disorders, whereas interferon gamma (IFN-γ) is elevated mainly in viral encephalitis [12, 13].

Quantifying metabolites in cerebrospinal fluid (CSF) is a promising approach to identify novel biomarkers for infectious and inflammatory CNS disorders, as CSF is in intimate contact with the target organ and has been evaluated as “liquid biopsy” for CNS pathology essentially since development of lumbar puncture in the nineteenth century. Moreover, small molecules can potentially be developed to rapid diagnostics that can be performed after lumbar puncture at the bedside or in an acute services lab and thus have the potential to provide shorter turn-around time than detection of pathogenic viruses and autoantibodies. We have previously applied a targeted metabolomics approach to measure 188 small molecules in CSF from 221 patients with infectious, autoimmune, and non-inflammatory CNS disorders. In previous publications we reported the identification of accurate biomarkers for CNS involvement in varicella-zoster virus reactivation [14], for enterovirus meningitis in patients without CSF pleocytosis [15], and for the differentiation between bacterial and viral meningitis [16, 17]. We have now expanded the analysis of this cohort to screen for CSF metabolite biomarkers that can accurately distinguish between viral CNS infections and autoimmune meuroinflammation and may reveal pathophysiological differences between the two.

## Methods

### Study cohort

We present a subtotal analysis of a cohort of CSF samples from 221 patients, which were collected between 2005 and 2013. The study was approved by the Ethics Committee of Hannover Medical School (file no. 2413–2014). Other findings in this cohort relating to CSF biomarkers have been described in [14–18]. For the current study, we analyzed data from CSF samples (N = 90) from patients with viral CNS infections (n = 34), consisting of herpes simplex virus encephalitis (HSE, n = 9), varicella zoster virus meningoencephalitis (VZV ME, n = 15), enterovirus meningitis (EntM, n = 10), autoimmune neuroinflammation (n = 25; multiple sclerosis, n = 17; autoimmune anti-NMDA-r encephalitis, n = 8), and non-inflamed controls (n = 31) comprising Bell’s palsy with normal CSF cell count (n = 11) and Gilles de la Tourette syndrome (for simplicity referred to as Tourette syndrome; GTS, n = 20). Diagnostic criteria (case definitions) are summarized in Additional file 1: Table S1.

### Standard clinical diagnostic parameters

The following standard CSF parameters were determined: cell count, protein concentration, lactate concentration, Q-albumin (CSF albumin/serum albumin), and IgG index (IgG ratio/Q-albumin). Blood-CSF-barrier (BCB) dysfunction was scored from 0 (no dysfunction) to 3 (severe dysfunction), using age-corrected Q-albumin as described in [19]. Leukocyte counts and C-reactive protein (CRP) levels in peripheral blood were determined at the time of lumbar puncture.

### Targeted metabolomics

CSF metabolite concentrations of the entire cohort were measured in 2015 in one batch using a triple-quadrupole mass spectrometer (API4000, Sciex, Framingham, MA, USA) with an electrospray-ionization ion source coupled to a high-performance liquid chromatography system (SIL-HTc, Shimadzu, Japan) and the AbsoluteIDQ™ p180 kit and MetIDQ™ software (Biocrates Life Sciences, Innsbruck, Austria) as described in detail in [14]. The following lipid nomenclature is used. Sphingomyelin (SM) and hydroxysphingomyelin (SM[OH]): Cx:y, where x = total number of carbon atoms and y = total number of double bonds in the amide bond. Phosphatidylcholine (PC): aa: both side chains are fatty acids linked to the glycerol backbone by ester bonds, ae: one of the side chains is a fatty alcohol linked to the glycerol backbone by an ether bond; Cx:y: x = total number of carbon atoms and y = total number of double bonds in both fatty acid chains.

### Quality screen

As in our recent study of CSF metabolite biomarkers for bacterial vs. viral CNS infections [17], we only included only those analytes that were detected > limit of detection (LOD = 3 × the signal obtained with the blank) in at least 80% of the sample group with the highest degree of neuroinflammation; in the present study this was the viral CNS infections group. This screen identified 85 analytes to be included in the downstream analyses. Specifically, there were 41 phosphatidylcholines (PC), 3 lysophosphatidylcholines (lysoPC), 9 sphingomyelins (SM), 18 amino acids (AA), 5 amino acid metabolites (AAM), 8 acylcarnitines (AC), and the sum of hexoses (Additional file 1: Fig. S1). All concentrations < LOD were replaced with the value LOD/2.

### Statistical analysis

Considering the non-normal distribution of the data, correlations were determined with the Spearman method, differences in median values between groups with the Mann–Whitney U test, and differences across groups with the Kruskall-Wallis test. Differences in categorical variables were assessed with the Chi – squared test. Biostatistical and bioinformatics analyses were carried out using R version 4.2.2. unless stated otherwise. Principal component analysis (PCA) was carried out with the R package "FactoMineR version 2.8" [20] and the PCA figure was made with the R package "factoextra version 1.0.7" [21]. The hierarchical clustering analyses shown in Fig. 2 and Additional file 1: Fig. S3 were performed with Metaboanalyst 5.0 (www.metaboanalyst.ca). R package “ROCR version 1.0.11” [22] was used for receiver operating characteristic (ROC) curve analysis. To calculate the area under the ROC curve (AUC) for a given marker, true positive rate (y-axis) and false positive rate (x-axis) are calculated at each cut-off value (based on the measured values for each analyte) and the AUC then corresponds to the area between the curve and the x-axis. An AUC of 1 indicates perfect classification, and an AUC of 0.5 indicates classification by chance alone. ROC analysis was repeated with 1000 bootstrap samples in order to compute the AUC confidence intervals (CI). Candidate biomarkers were then subjected to leave-one-out (jackknife) internal cross validation [23, 24]. We calculated how many times a feature was selected in all iterations of the cross-validation within the subset of the 10 biomarker candidates with the highest AUC, using the frequency of selection as a measure of robustness of a biomarker. High AUC Abundance (HAUCA) curves were constructed as described previously [25]. Sensitivity, specificity, positive predictive value (PPV), negative predictive value (NPV) and the corresponding cut-off values were calculated using the Youden index method and R package “OptimalCutpoints version 1.1.5” [26].

## Results

### Study cohort

Sociodemographic and clinical data of the three groups are summarized in Table 1. Additional file 1: Table S2 provides the same data for each of the 7 underlying diagnostic groups. Consistent with the epidemiology of autoimmune disorders, this group had a somewhat lower mean age and a preponderance of females. As expected, CSF cell count, lactate levels, and BCB disruption were highest in viral CNS infections, whereas IgG index was highest in autoimmune disorders. Thus, the study groups reflect the natural history of the underlying disorders.

**Table 1:** Demographic and clinical laboratory characteristics

	Parameter	CNS viral infections n = 34^a^	Autoimmune neuroinflammation n = 25^b^	Controls n = 31^c^	P value
		Median (range)			
	Age (years)	47 (13–80)	33 (19–69)	40 (19–83)	0.03^d^
	Sex Male %	65	40	74	0.018^e^
	Female %	35	60	26	
Blood	Leukocyte count (1000/µL)	7.3 (1.8–14)	6.7 (2.1–18.2)	6.8 (4.6–11.9)	0.69^d^
	C-reactive protein (mg/L)	3 (1–128)	1.8 (1–37)	3 (1–31)	0.71^d^
CSF	Cell count (1/µl)	37.7 (0.7–1536)	7.85 (0.7–172)	1.3 (0.3–4.7)	1.6e-10^d^
	Lactate (mmol/L)	2.2 (1.5–5.5)	1.8 (1.3–2.6)	1.6 (1.2–2.2)	8.3e-07^d^
	Protein (mg/L)	0.65 (0.24–2.27)	0.47 (0.24- 0.88)	0.4 (0.23–0.83)	5.4e-06^d^
	Q IgG	5.96 (1.3–58.4)	4.85 (1.6–13.2)	2.4 (1.3–6.5)	2e-08^d^
	IgG Index	0.57 (0.47–1.3)	0.75 (0.53–2.9)	0.5 (0.41–1)	1.7e-8^d^
	Q albumin	11.4 (2.7–44.4)	5.84 (2.5–12.3)	4.7 (2.4–14)	7.5e-09^d^
	BCB disruption (%)				
	None (1)	18	60	71	0.0005^e^
	Light (2)	40	40	29	
	Moderate (3)	18	0	0	
	Severe (4)	24	0	0	

^a^Comprising HSV encephalitis (n = 9), VZV meningitis/encephalitis (n = 15), enterovirus meningitis (n = 10)

^b^Comprising anti-NMDA receptor autoimmune encephalitis (n = 8) and multiple sclerosis (n = 17)

^c^Comprising Tourette Syndrome (n = 20), Bell’s palsy (n = 11)

^d^Kruskal—Wallis test ^e^ Chi—squared test

### CSF metabolite populations differ between viral CNS infections and autoimmune neuroinflammation

**Fig. 1:**
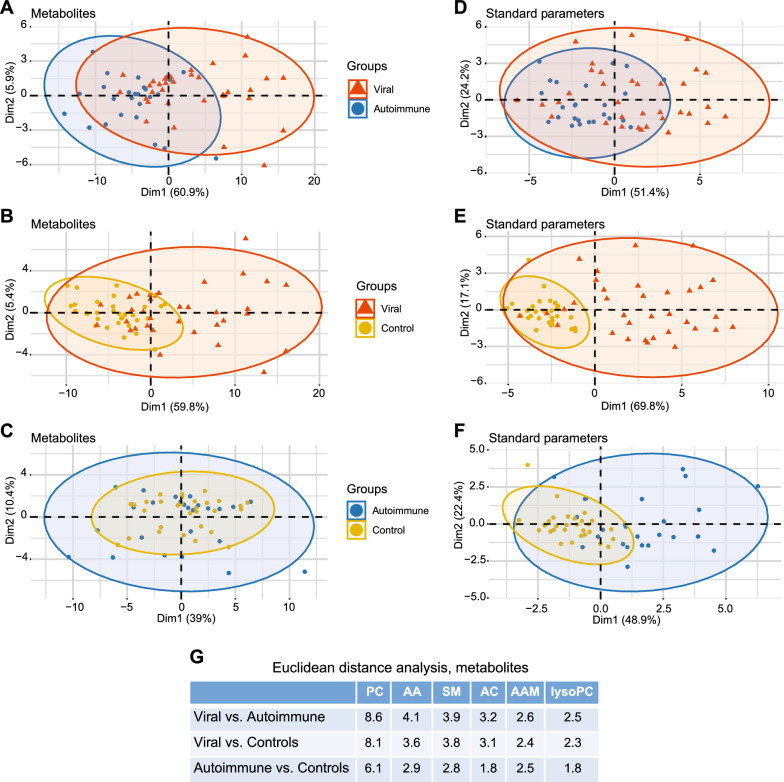
Global differences in CSF metabolite populations between viral CNS infections and autoimmune neuroinflammation or noninflamed controls are mostly driven by changes in free phospolipids. **A-C**. PCA was performed on the basis of 85 CSF analytes: 41 phosphatidylcholines (PC), 3 lyso phosphatidylcholines (lysoPC), 9 sphingomyelins (SM), 18 amino acids (AA), 5 amino acid metabolites (AAM), 8 acylcarnitines (AC), and 1 generic hexose. **A**. Viral CNS infections vs. autoimmune neuroinflammation. **B**. Viral CNS infections vs. controls. **C**. Autoimmune neuroinflammation vs. controls. **D-F**. PCA was performed on the basis of 7 standard diagnostic CSF parameters (leukocyte count, lactate, protein, BCB disruption, Q albumin, IgG Index, Q IgG). **D**. Viral CNS infections vs. autoimmune neuroinflammation **E**, Viral CNS infections vs. controls **F**, Autoimmune neuroinflammation vs. controls. **G**. Mean Euclidian distances (reflecting global changes in individual metabolite classes) between viral CNS infections, autoimmune neuroinflammation, and controls. Group sizes: viral CNS infections, *n* = 34; autoimmune neuroinflammation, *n* = 25; controls, *n* = 31

**Fig. 2:**
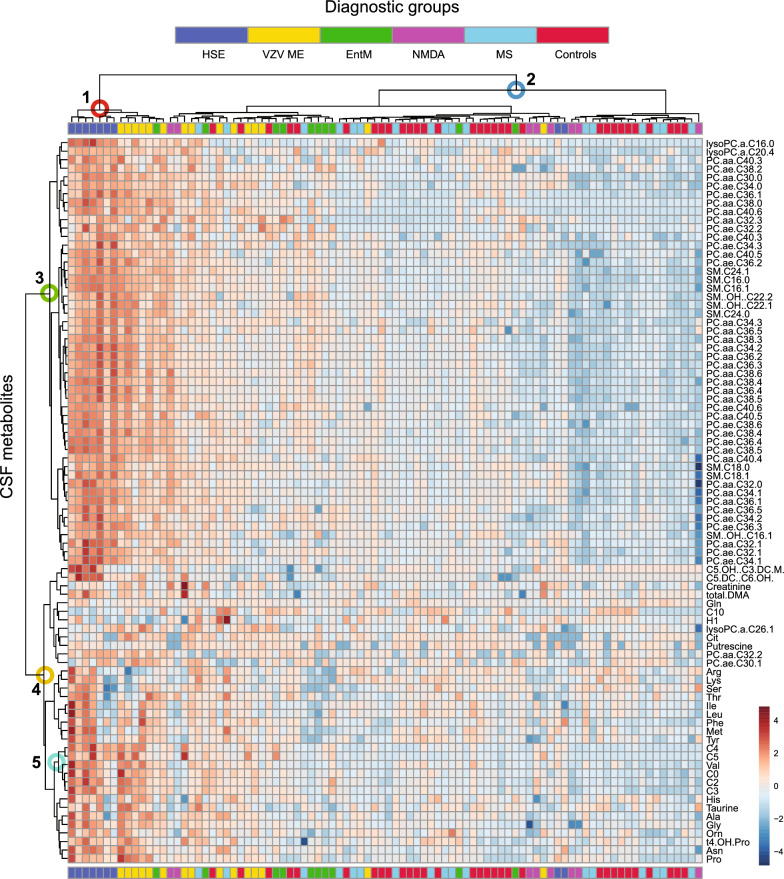
Patient classification based on CSF metabolite concentrations. Unsupervised hierarchical biclustering analysis using the same metabolite data as for Fig. 1. The samples are clustered along the x-axis, and the diagnostic groups are identified by the color code in the legend. Metabolites are clustered along the y-axis. The numbered nodes identify the clades with the following features: 1 (red circle), viral CNS infections, predominantly HSE and VZV ME; 2 (dark blue circle) controls, autoimmune neuroinflammation, and less inflamed viral samples, e.g. enterovirus meningitis. 3 (green circle), phospholipids; 4 (yellow circle) amino acid, amino acid metabolites, and acylcarnitines; 5 (light blue circle), short-chain acylcarnitines and amino acids. HSE = HSV encephalitis; VZV ME = VZV meningoencephalitis; EntM = enterovirus meningitis; MS = multiple sclerosis, NMDA = anti-NMDAr autoimmune encephalitis

We used principle component analysis (PCA) to obtain an overview over differences in CSF metabolites among the three groups. Indeed, there was a tendency toward separation between viral CNS infections and autoimmune neuroinflammation, which was about as pronounced as the separation between viral CNS infections and controls, whereas autoimmune neuroinflammation and controls overlapped considerably (Fig. 1A-C). For all three comparisons, scree plots show that principle component 1, which contributes most to variance, is driven by alterations in phospholipid concentrations, whereas component 2 is driven by differences in amino acids and, in the case of viral CNS infections vs. autoimmune neuroinflammation or controls, short chain acylcarnitines C0 and C3 (Additional file 1: Figure S2). Consistent with this relatively uncoupled regulation of phospholipids from amino acids and acylcarnitines, a correlation matrix of all metabolites across all samples revealed a large clade of phospholipids that correlated positively among each other, whereas amino acids and acyl carnitines clustered in other clades characterized by weaker positive or even negative correlations, and very few phospholipids (Additional file 1: Figure S3). Compared to the metabolites, separation based on the standard parameters appeared to be less clear for viral CNS infections vs. autoimmune neuroinflammation, but better for viral CNS infections and autoimmune neuroinflammation vs. controls (Fig. 1D-F). Confirming the pronounced differences between viral CNS infections and autoimmune neuroinflammation, analysis of mean Euclidian distances showed that, among the three sample groups, the greatest distances based on metabolite concentrations were between viral CNS infections and autoimmune neuroinflammation for all 6 metabolite subgroups (Fig. 1G). A hierarchical clustering analysis (Fig. 2) grouped the patient samples into two major clades. One clade (marked 1, red circle) consisted of 7 HSE and 6 VZV ME, and 1 EntM sample and was characterized by elevated CSF concentrations of phospholipids (metabolite clade marked 3, green circle) and short-chain acylcarnitines (C0, C2-C5) and valine (metabolite subclade marked 5, light blue circle on y-axis), which was contained within a larger metabolite clade (marked 4, yellow circle) comprised predominantly of amino acids, amino acid metabolites, and acylcarnitines. The other major clade (marked 2, dark blue circle) contained the control samples and autoimmune neuroinflammation samples and, mostly in a distinct subclade, the remaining viral samples. Taken together, these results suggested a high potential of the CSF metabolites to differentiate between viral CNS infections and autoimmune neuroinflammation or controls, but not between autoimmune neuroinflammation and controls.

### Biomarker identification

Considering the lack of an external validation cohort, we performed a HAUCA curve analysis to estimate the likelihood of identifying false positive biomarkers by ROC analysis (Additional file 1: Fig. S4). It turned out to be low for the comparisons viral CNS infections vs. autoimmune neuroinflammation and viral CNS infections vs. controls, as the upper CIs of the random datasets did not cross the lines defined by the real datasets. In contrast, the potential for identifying false positive biomarkers to differentiate between autoimmune neuroinflammation and controls was very high, as these lines crossed extensively.

**Fig. 3:**
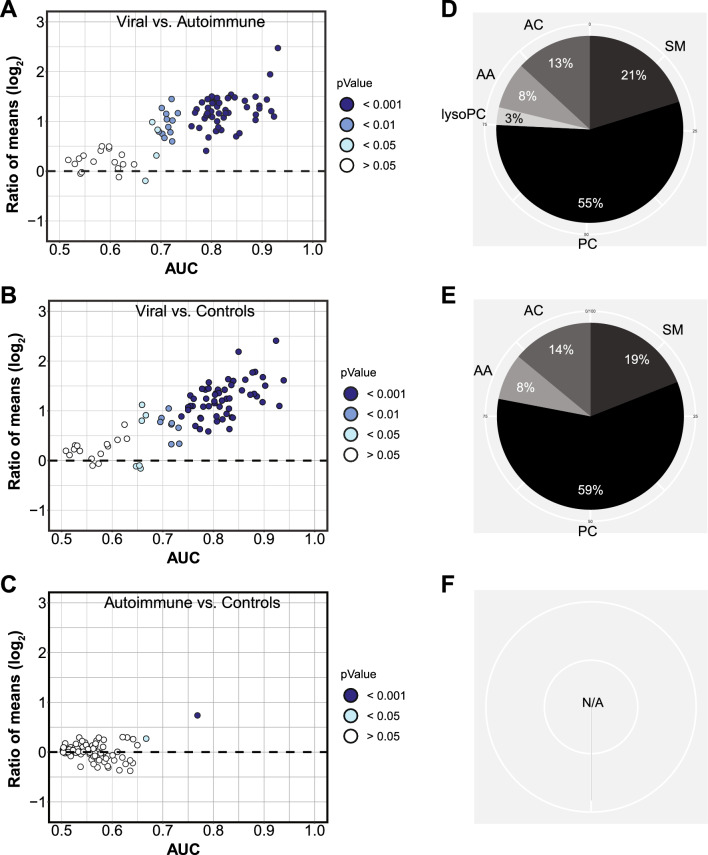
CSF metabolites yield accurate biomarkers for the differentiation between viral CNS infections and autoimmune neuroinflammation. **A-C**. Dispersion plots based on binary receiver operating characteristic (ROC) curve analysis of the same 85 CSF metabolites as used for Fig. 1 and 2. For each pairwise comparison, ratio of mean concentrations (“fold change”) is plotted on the y-axis, area under the ROC curve (AUC) along the x-axis; each circle represents one metabolite, with the fill color indicating asymptotic significance of the ROC curve. **A**. Viral CNS infections vs. autoimmune neuroinflammation.** B**. Viral CNS infections vs. controls**. C**. Autoimmune neuroinflammation vs controls. **D-F**, Percentages of all accurate biomarkers (AUC > 0.8, P < 0.05, lower CI > 0.5) that originate from each of the 6 metabolite subclasses. **D,** Viral CNS infections vs. autoimmune neuroinflammation. **E**, Viral CNS infections vs. controls. **F,** Autoimmune neuroinflammation vs. controls (N/A = not applicable, as all AUCs were < 0.8)

We then applied ROC curve analysis to screen the metabolite data set for accurate biomarker candidates (Fig. 3). We defined accurate biomarker candidates as having an AUC > 0.8 (indicating “excellent classification” according to the Hosmer and Lemshow grading [27]), an asymptotic *p* value ≤ 0.05, and the lower CI not crossing 0.5. Thirty-eight of the 85 analytes (45%) fulfilled these criteria for the differentiation between viral CNS infections vs. autoimmune neuroinflammation, and 37 (44%) for the differentiation between viral CNS infections vs. controls. There were no accurate biomarkers for autoimmune neuroinflammation vs. controls, as the highest AUC was 0.77. Consistent with the heat map shown in Fig. 2, concentrations of all biomarker candidates were higher in viral CNS infections than in the other groups and comprised predominantly phosphatidylcholines, followed by sphingomyelins and acylcarnitines (Fig. 3, Additional file 1: Table S3). The 10 most robust biomarkers were then selected by leave-one-out internal cross-validation and ranked according to AUC (Table 2, Additional file 1: Table S4, S5). As suggested by the heat map, these markers comprised mostly phospholipids, but, notably, short-chain acylcarnitines C4 (isobutyrylcarnitine) and C5 (isovalerylcarnitine) were among the top 4 biomarkers for viral CNS infections vs. autoimmune neuroinflammation. Concentrations of these acylcarnitines also correlated strongly which each other (correlation matrix in Additional file 1: Figure S3). These 10 most accurate metabolite biomarkers had higher AUCs (0.89–0.93) than the best standard CSF parameter, Q albumin (AUC = 0.86). Cell count was the most accurate (AUC = 0.93) standard parameter biomarker for the distinction between viral CNS infections vs. controls: it was about as accurate as the top 10 metabolite biomarkers, which comprised glycerophospholipids, the amino acid glycine, and acylcarnitines C4 and C5 (Additional file 1: Table S4). Importantly, while there were no accurate metabolite biomarkers to distinguish between autoimmune neuroinflammation and controls, our analysis validated IgG index as accurate (AUC = 0.90) diagnostic standard CSF parameter for this distinction (Additional file 1: Table S5).

**Table 2:** Comparison between standard diagnostic CSF and blood parameters and the 10 most accurate CSF metabolite biomarkers to differentiate between viral CNS infections and autoimmune neuroinflammation

Standard parameters			CSF metabolites			
Parameter	AUC (95% CI)	Ratio of means	Metabolite	AUC (95% CI)	Ratio of means	Selection frequency^a^
Q albumin	0.86*** (0.76–0.95)	2.7	C5	0.93*** (0.87–0.99)	5.6	1
BCB disruption	0.78*** (0.67–0.88)	n/a	PC.aa.C30.0	0.92*** (0.84–1.0)	2.1	1
CSF protein	0.77*** (0.65–0.88)	1.9	PC.ae.C32.2	0.92*** (0.85–0.99)	2.3	1
CSF lactate	0.74** (0.62–0.86)	1.3	C4	0.92*** (0.85–0.98)	3.9	1
CSF cell count	0.74** (0.61–0.86)	8.7	SM.C16.0	0.91*** (0.83–0.99)	2.7	1
IgG-index	0.73** (0.60–0.86)	0.63	PC.aa.C38.0	0.89*** (0.82–0.97)	2.5	1
Q IgG	0.67* (0.53–0.81)	1.9	PC.ae.C34.0	0.89*** (0.81–0.98)	2.4	1
Blood CRP	0.55 (0.40–0.69)	2.1	PC.ae.C36.1	0.89*** (0.81–0.97)	2.2	1
Blood leukocytes	0.54 (0.39–0.70)	1.1	SM.C16.1	0.89*** (0.80–0.97)	2.8	1
			PC.aa.C40.6	0.89*** (0.78–0.98)	2	0.98

^a^Frequency of selection in leave-one-out cross-validation. 1 = always selected, 0 = never selected. ** *p* ≤ 0.01; *** *p* ≤ 0.001

Using trade-off values in the ROC curves at the maximal Youden index values, we then determined sensitivity, specificity, and positive and negative predictive value (PPV, NPV) of the same standard parameters and the six CSF metabolites with the highest AUC for the differentiation between viral CNS infections and autoimmune neuroinflammation (Table 3). Even though there were highly sensitive (e.g., Qalb) or specific (e.g., IgG index, BCB disruption) standard parameters, the metabolites differed in that they had both high sensitivity and specificity. Indeed, all 6 metabolites had higher Youden index values (sensitivity + specificity—1) than the best standard parameter. Similar differences between standard parameters and metabolites were seen regarding PPV and NPV. Sensitivity, specificity, PPV and NPV of the differentiation between viral CNS infections or autoimmune neuroinflammation and controls are summarized in Additional file 1: Tables S6 and S7, respectively.

**Table 3:** Comparison of common blood and CSF standard diagnostic parameters and the top six CSF metabolite biomarkers: sensitivity, specificity, PPV, and NPV for viral CNS infections vs. autoimmune neuroinflammation^a^

Biomarkers		Sensitivity	Specificity	PPV	NPV	Cut-off value	Youden index
Blood	Leukocytes	0.36	0.83	0.75	0.49	9.8 (1000/µL)	0.19
	CRP	0.67	0.5	0.67	0.5	2 mg/L	0.17
CSF Standard parameters	Qalb	0.97	0.6	0.76	0.94	6.3	0.57
	CSF cell count	0.74	0.75	0.81	0.67	15.3 1/µl	0.49
	CSF protein	0.48	0.96	0.94	0.58	0.68 mg/L	0.44
	BCB disruption	0.42	1	1	0.57	3	0.42
	CSF lactate	0.82	0.57	0.73	0.68	1.8 mmol/L	0.39
	Q IgG	0.33	1	1	0.52	13.6	0.33
	IgG Index	1	0	0.58	–	0.47	0.0
CSF Metabolites	C5	0.94	0.84	0.89	0.91	0.02 µM	0.78
	PC.aa.C30.0	0.94	0.84	0.89	0.91	0.06 µM	0.78
	PC.ae.C32.2	0.88	0.88	0.91	0.85	0.01 µM	0.76
	SM.C16.0	0.94	0.80	0.86	0.91	0.43 µM	0.74
	PC.aa.C38.0	0.85	0.88	0.91	0.81	0.011 µM	0.73
	C4	0.91	0.80	0.86	0.87	0.03 µM	0.71

^a^Within each class (blood parameters, standard CSF parameters, CSF metabolites), markers are ranked by Youden index (sensitivity + specificity—1 at the optimal cut-off point in the ROC curve) in descending order

### CSF concentrations of the top 6 biomarkers

**Fig. 4:**
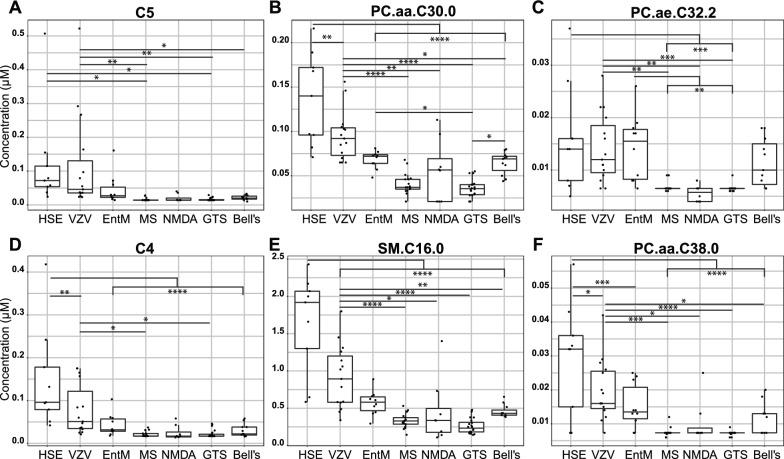
Concentrations of the six most accurate CSF metabolite biomarkers across the diagnostic subgroups. CSF concentrations based on the same LC–MS/MS dataset used for the other analyses of this report. **A**, C5. **B**, PC.aa.C30.0. **C,** PC.ae.C32.2. **D**, C4. **E**, SM.C16.0. **F**, PC.aa.C38.0. HSE = HSV encephalitis; VZV = VZV meningoencephalitis; EntM = enterovirus meningitis; MS = multiple sclerosis, NMDA = anti-NMDAr autoimmune encephalitis, GTS = Tourette syndrome, Bell’s = Bell’s palsy. One-way ANOVA with Tukey’s post-hoc test. * *p* ≤ 0.05; ** *p* ≤ 0.01; *** *p* ≤ 0.001

We then tested whether concentrations of the top 6 biomarkers differed by diagnostic subgroup within each of the three supergroups (Fig. 4). Among the three viral etiologies, median concentrations of 4 biomarkers were clearly highest in HSV encephalitis, the viral CNS infection with the greatest risk of cell damage and long-term sequelae, supporting a role in target cell and end-organ damage. On the other hand, the lowest concentrations of all 6 biomarkers among the three viral subgroups were measured in enterovirus meningitis, which features the lowest clinical risk of severe disease and long-term complications. The lowest concentrations across all groups were generally found in autoimmune neuroinflammation and controls. However, within the control group, there was a notable tendency toward higher concentrations of the 4 phospholipids in Bell’s palsy than in Tourette syndrome. There was a higher percentage of female participants with Bell’s palsy than with Tourette syndrome (Additional file 1: Table S2). However, the higher phospholipid values in Bell’s palsy could not be explained by sex-specific differences between the two groups, as only PC.aa.C30.0 levels were significantly higher in samples from female than from male patients with Bell’s palsy (Additional file 1: Table S8).

### Correlation with CNS inflammation

**Fig. 5:**
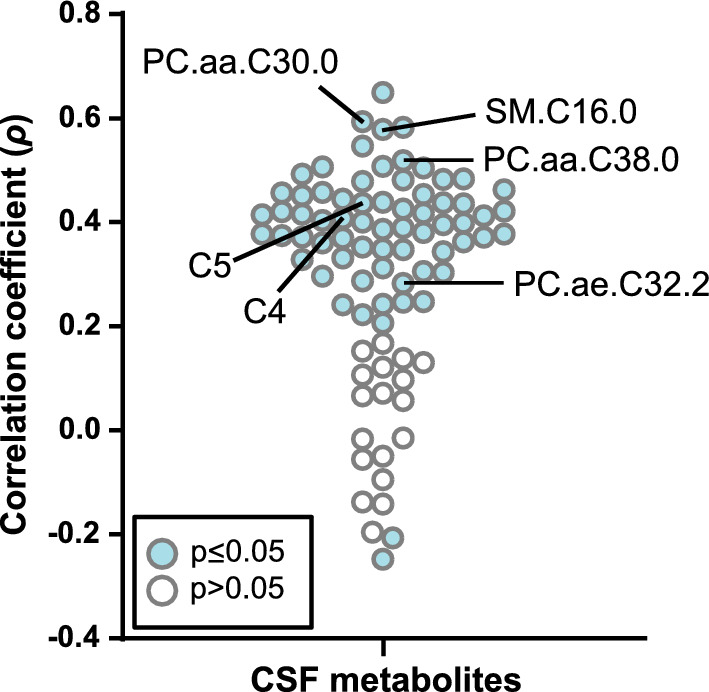
Correlations between CSF metabolites and CSF cell count. Correlations between concentrations of the 85 CSF metabolites and CSF cell count were determined by Spearman correlation. Correlation coefficients (*ρ*) are plotted along the y-axis. The black lines identify the six best validated metabolite biomarkers shown in Fig. 4. Numeric values: PC.aa.C30.0 (*ρ* = 0.59, *p* = 1.7 × 10^–5^); SM.C16.0 (0.58, 2.3 × 10^–9^); PC.aa.C38.0 (0.52; 1.6 × 10^–7^); C5 (0.44; 1.7 × 10^–5^); C4 (0.41; 6.5 × 10^–5^); PC.ae.C32.2 (0.28, 7 × 10^–3^)

We then tested to what extent concentrations of CSF metabolites across all samples correlated with CSF leukocyte count as a measure of neuroinflammation (Fig. 5). Most correlations were positive, but correlation coefficient values (*ρ*) were modest, ranging from -0.25 (arginine) to 0.65 (proline). The correlation coefficients of the six top biomarkers ranged from 0.28 to 0.58, indicating that the observed changes in their concentrations were only partially driven by CNS inflammation and may, for instance, reflect alterations in CNS resident cells.

## Discussion

We performed a targeted metabolomic analysis of CSF samples from patients with viral CNS infections, autoimmune neuroinflammation, and noninflamed controls and found that CSF metabolites were more accurate biomarkers than standard diagnostic CSF parameter for the differentiation between viral CNS infections and autoimmune neuroinflammation, whereby the short-chain acylcarnitines C4 (isobutyrylcarnitine) and C5 (isovalerylcarnitine) were among the best metabolite biomarkers.

Acylcarnitines are intermediates of beta-oxidation, i. e. the metabolism of fatty acids to acetyl-CoA, which is fed into the tricarboxylic acid cycle as energy source and building block. Elevated extracellular levels of short-chain acylcarnitines usually reflect a dysfunction of beta-oxidation of saturated fatty acids in mitochondria, which leads to the conjugation of accumulating fatty acid fragments to the carrier molecule carnitine. These acylcarnitine complexes are then extruded into the cytoplasm and subsequently into the extracellular environment [28]. In our study we noted that the highest C4 and C5 concentrations in CSF were found in HSE, which is known to interfere considerably more strongly with cell homeostasis (and cause organ damage) than VZV or enteroviruses. A plausible explanation would, therefore, be that the viruses, and particularly HSV, compromise mitochondrial utilization of fatty acids in the CNS, favoring glycolysis as a readily available energy source. This is also supported by higher lactate concentrations in viral CNS infections than autoimmune neuroinflammation (Table 2). There also is experimental evidence that both HSV and VZV induce mitochondrial dysfunction in human cells [29, 30]. This mitochondrial dysfunction may also result from interferon responses triggered by these viruses. Deleting interferon-γ stimulated gene 15 (ISG15) in human cells results in hyperactive interferon signaling, mimicking a viral infection. ISG15-deficient cells have an interferon-driven defect in mitochondrial respiration, which leads to compromised fatty acid oxidation and elevated acylcarnitine levels [31, 32], and pharmacologic inhibition of interferon signaling leads to a normalization of fatty acid oxidation in the ISG15-deficient cells [32].

All other accurate biomarkers for viral CNS infections vs. autoimmune neuroinflammation were membrane phospholipids, and their concentrations were always higher in viral CNS infections. This agrees well with our previous observations that, even though the highest phospholipid levels in CSF are found in bacterial meningitis [17], elevated concentrations of specific phospholipid species are also found in viral infections, as compared to noninflamed control samples [14, 15]. Viruses interact with host cell membranes in complex ways. Cell damage in the sense of a cytopathic effect would be the simplest explanation for the release of membrane phospholipids during viral infections, and this agrees well with our observation that levels were highest in HSE. However, more subtle mechanisms such as differences in membrane signaling or perturbations during viral entry and budding are also plausible.

Using LC–MS/MS analysis of the same cohort, we have previously reported that CSF kynurenine and the tryptophan/kynurenine ratio constitute biomarkers for the differentiation between viral CNS infections and autoimmune neuroinflammation [18], which was subsequently corroborated using a simple immunoassay for these analytes [33]. Kynurenine was studied in [18] because it had been detected > LOD preferentially in infected samples and therefore held promise as a biomarker for infectious CNS processes, but it was excluded from the present analysis because it was detected > LOD in < 80% of the samples. The AUCs for kynurenine and tryptophan/kynurenine ratio in [18] for the differentiation between viral CNS infections and autoimmune neuroinflammation were 0.8 and 0.79, respectively, which is substantially lower than the AUCs of the CSF metabolite biomarkers identified in the present study. However, the AUCs for both kynurenine and tryptophan/kynurenine ratio increased to 0.95 when CSF cell count was added in a logistic regression model. Future studies should investigate whether logistic regression models containing selected CSF metabolite biomarkers described herein and standard CSF parameters result in a similar improvement of diagnostic potential.

The diagnosis of Bell’s palsy is made in individuals with acute onset of paralysis of the facial nerve after excluding known causes of facial neuritis including infections with, e.g., *Borrelia* sp. or VZV [34]. Major risk factors are diabetes, hypertension, pregnancy, and obesity. In the Bell’s palsy patients in our cohort, common infectious etiologies had been excluded. However, concentrations of certain CSF metabolite biomarkers for viral CNS infections was slightly higher than in the Tourette syndrome controls, suggesting an inflammatory etiology even though CSF cell counts were normal in all Bell’s palsy samples. This also agrees with our previous observation that phospholipid levels are elevated in CSF samples from enterovirus meningitis with normal cell counts [15]. Taken together, these results support the common concept that Bell’s palsy often has an inflammatory origin, such as post-infectious processes after infections with pathogens that are not usually tested for in clinical practice.

### Limitations and strengths of the study

Our study is limited in that the samples were collected at a single center and we could, therefore, not perform an external validation, which would provide stronger evidence for the validity of the biomarkers. The results should, therefore, be replicated in an independent cohort before further development to a clinical diagnostic can be considered. In addition, the exact molecular identity of some of the identified phosphatidylcholines needs to be defined further, as some of them correspond to more than one isobar or isomer. This metabolomics analysis targeted only a selection of metabolite classes, and other markers for mitochondrial function (e.g., tricarboxylic acid intermediates, cytochrome C, ATP, NADH) or membrane perturbation (e.g., ceramides) were not included. The Human Metabolome Database (hmdb.ca) lists 468 endogenous CSF metabolites, and a broader screen might detect additional biomarkers. On the other hand, a clear strength of the study is that all specimens were collected using a standardized protocol and that definitive diagnoses according to viral pathogen and inflammatory disorder were available.

### Supplementary Information

**Additional file 1: Fig. S1.** Quality screen used to identify analytes to be included in the analysis. Analytes were included that were detected ≥ LOD in ≥ 80% of all samples. The numbers on top of the bars state the number of analytes that passed this screen divided by the total number of analytes in the respective metabolite subgroup. Detection efficiency was highest for amino acids, but phosphatidylcholines constituted the largest group of included analytes. Abbreviations: AA, amino acids; AAM, amino acid metabolites; AC, acylcarnitines; lysoPC, lysophosphatidylcholines; PC, phosphatidylcholines; SM, sphingomyelins; Hex, sum of hexoses. **Fig. S2.** Phospholipids make the greatest contributions to variance in the CSF metabolite populations. A, C, E. Scree plots illustrating the contribution of each dimension to overall variance in 85 metabolites in the PCA shown in Fig. 1. A, Viral CNS infections vs. autoimmune neuroinflammation. C, Viral CNS infections vs. controls. E, Autoimmune neuroinflammation vs. controls. B, D, F. Contributions of each feature to variance in the 1st and 2nd dimension in the PCA. B, Viral CNS infections vs. autoimmune neuroinflammation. D, Viral CNS infections vs. controls. F, Autoimmune neuroinflammation vs. controls. **Fig. S3.** Correlations among concentrations of the 85 CSF metabolites across all samples. Correlations among all analytes were determined using Pearson correlation analysis. The resulting Pearson correlation coefficients (*ρ)* were then used as input into a hierarchical clustering analysis. The correlation coefficient values are indicated by the color scheme shown in the legend. The arrows point to short-chain acylcarnitines C4 and C5. **Fig. S4.** HAUCA curve analysis to assess the likelihood of false positive biomarker identification. The number of biomarkers exceeding a given AUC value in ROC analysis is plotted on the x-axis. The analysis compares the number of biomarkers identified in the real data set (blue curve) to those identified in a random data set of the same variance (black curve), indicating the number of biomarkers expected by chance alone. The red curve delineates the upper bound 95% CI of the random data set. An AUC of 0.8 was used as AUC cut-off to define accurate biomarkers in the ROC curve analysis shown in Fig. 3. A. Viral CNS infections vs. autoimmune neuroinflammation. No markers in the random data set are expected to exceed AUC = 0.8, but 38 did so in the real data set. B. Viral CNS infections vs. controls. No markers in the random data set are expected to exceed AUC = 0.8, but 37 did so in the real data set. C. Autoimmune neuroinflammation vs. controls. No markers in the random or the real data set exceed AUC = 0.8.** Table S1. **Diagnostic criteria and clinical information. **Table S2. **Demographic and clinical laboratory characteristics of the seven subgroups. **Table S3:** Differences among the metabolite classes in biomarker potential. **Table S4.** Comparison of standard parameters and the 10 most robust CSF metabolite biomarkers to differentiate between viral CNS infections and controls. **Table S5.** Comparison of standard parameters and the 10 most robust CSF metabolite biomarkers to differentiate between autoimmune neuroinflammation and controls. **Table S6.** Comparison of common blood and CSF standard diagnostic parameters and the top five CSF metabolite biomarkers: sensitivity, specificity, PPV, and NPV for viral CNS infections vs. controls. **Table S7.** Comparison of common blood and CSF standard diagnostic parameters and the top five CSF metabolite biomarkers: sensitivity, specificity, PPV, and NPV for autoimmune neuroinflammation vs. controls. **Table S8. **Sex-specific concentrations in Bell’s palsy CSF samples of the top 6 CSF metabolite biomarkers for the differentiation between viral CNS infections and autoimmune inflammation.

## Data Availability

The data used and/or analyzed are available at https://figshare.com, 10.6084/m9.figshare.24321475.

## Abbreviations

AUC: Area under the curve
AC: Acylcarnitine
BCB: Blood-cerebrospinal-fluid barrier
C4: Isobutyrylcarnitine
C5: Isovalerylcarnitine
CI: Confidence interval
CNS: Central nervous system
CRP: C-reactive protein
CSF: Cerebrospinal fluid
EntM: Enterovirus meningitis
GTS: Gilles de la Tourette syndrome
HAUCA: High AUC Abundance
HSE: Herpes simplex virus encephalitis
HSV: Herpes simplex virus
ISG15: Interferon-γ stimulated gene 15
LC–MS/MS: Liquid chromatography tandem mass-spectrometry
LOD: Limit of detection
lysoPC: Lysophosphatidylcholine
ME: Meningoencephalitis
NMDA: N-methyl-D-aspartate
NPV: Negative predictive value
PC: Phosphatidylcholine
PCA: Principle component analysis
PPV: Positive predictive value
ROC: Receiver operating characteristic
SM: Sphingomyelin
VZV: Varicella zoster virus
