# Supplementary material for: Elevated phospholipids and acylcarnitines C4 and C5 in cerebrospinal fluid distinguish viral CNS infections from autoimmune neuroinflammation
Source: J Transl Med. 2023 Nov 2;21:776. doi: 10.1186/s12967-023-04637-y (PMC10621113; doi:10.1186/s12967-023-04637-y)
Supplement: Supplementary file 1 — Additional file 1: Fig. S1. Quality screen used to identify analytes to be included in the analysis. Analytes were included that were detected ≥ LOD in ≥ 80% of all samples. The numbers on top of the bars state the number of analytes that passed this screen divided by the total number of analytes in the respective metabolite subgroup. Detection efficiency was highest for amino acids, but phosphatidylcholines constituted the largest group of included analytes. Abbreviations: AA, amino acids; AAM, amino acid metabolites; AC, acylcarnitines; lysoPC, lysophosphatidylcholines; PC, phosphatidylcholines; SM, sphingomyelins; Hex, sum of hexoses. Fig. S2. Phospholipids make the greatest contributions to variance in the CSF metabolite populations. A, C, E. Scree plots illustrating the contribution of each dimension to overall variance in 85 metabolites in the PCA shown in Fig. 1. A, Viral CNS infections vs. autoimmune neuroinflammation. C, Viral CNS infections vs. controls. E, Autoimmune neuroinflammation vs. controls. B, D, F. Contributions of each feature to variance in the 1st and 2nd dimension in the PCA. B, Viral CNS infections vs. autoimmune neuroinflammation. D, Viral CNS infections vs. controls. F, Autoimmune neuroinflammation vs. controls. Fig. S3. Correlations among concentrations of the 85 CSF metabolites across all samples. Correlations among all analytes were determined using Pearson correlation analysis. The resulting Pearson correlation coefficients (ρ) were then used as input into a hierarchical clustering analysis. The correlation coefficient values are indicated by the color scheme shown in the legend. The arrows point to short-chain acylcarnitines C4 and C5. Fig. S4. HAUCA curve analysis to assess the likelihood of false positive biomarker identification. The number of biomarkers exceeding a given AUC value in ROC analysis is plotted on the x-axis. The analysis compares the number of biomarkers identified in the real data set (blue curve) to those identifie [file 12967_2023_4637_MOESM1_ESM.docx]

**Supplement**

**Al-Mekhlafi et al.**

**Table of Contents**

**Item**

Figure S1

Figure S2

Figure S3

Figure S4

Table S1

Table S2

Table S3

Table S4

Table S5

Table S6

Table S7

Table S8

**Fig. S1. Quality screen used to identify analytes to be included in the analysis.** Analytes were included that were detected ≥LOD in ≥80% of all samples. The numbers on top of the bars state the number of analytes that passed this screen divided by the total number of analytes in the respective metabolite subgroup. Detection efficiency was highest for amino acids, but phosphatidylcholines constituted the largest group of included analytes. Abbreviations: AA, amino acids; AAM, amino acid metabolites; AC, acylcarnitines; lysoPC, lysophosphatidylcholines; PC, phosphatidylcholines; SM, sphingomyelins; Hex, sum of hexoses.


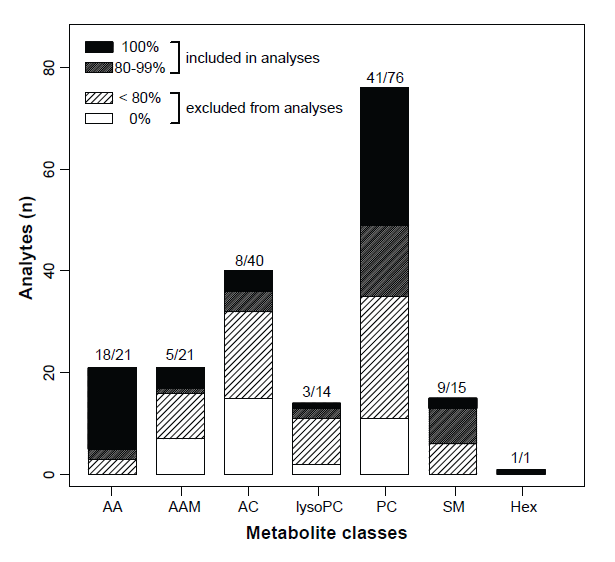


**Fig. S2. Phospholipids make the greatest contributions to variance in the CSF metabolite populations. A**, **C**, **E**. Scree plots illustrating the contribution of each dimension to overall variance in 85 metabolites in the PCA shown in Fig. 1. **A**, Viral CNS infections vs. autoimmune neuroinflammation. **C**, Viral CNS infections vs. controls. **E,** Autoimmune neuroinflammation vs. controls. **B**, **D**, **F**. Contributions of each feature to variance in the 1^st^ and 2^nd^ dimension in the PCA. **B**, Viral CNS infections vs. autoimmune neuroinflammation. **D**, Viral CNS infections vs. controls. **F,** Autoimmune neuroinflammation vs. controls.


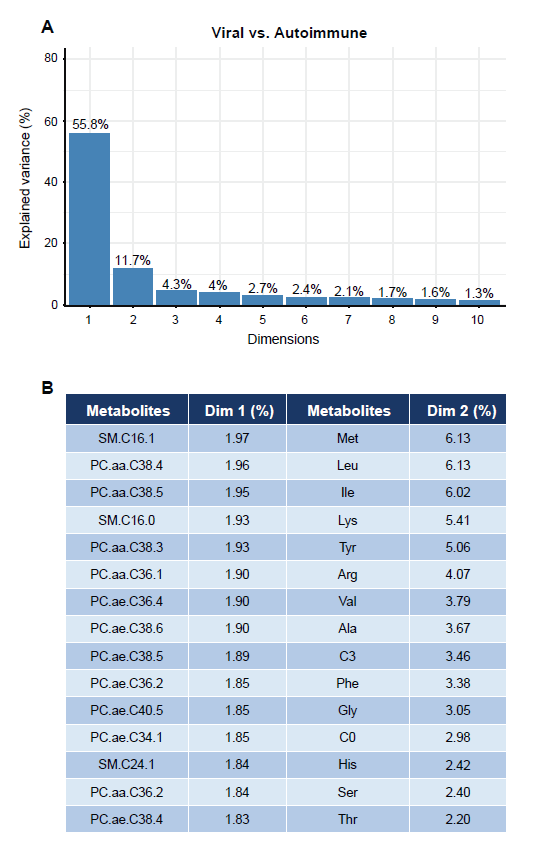


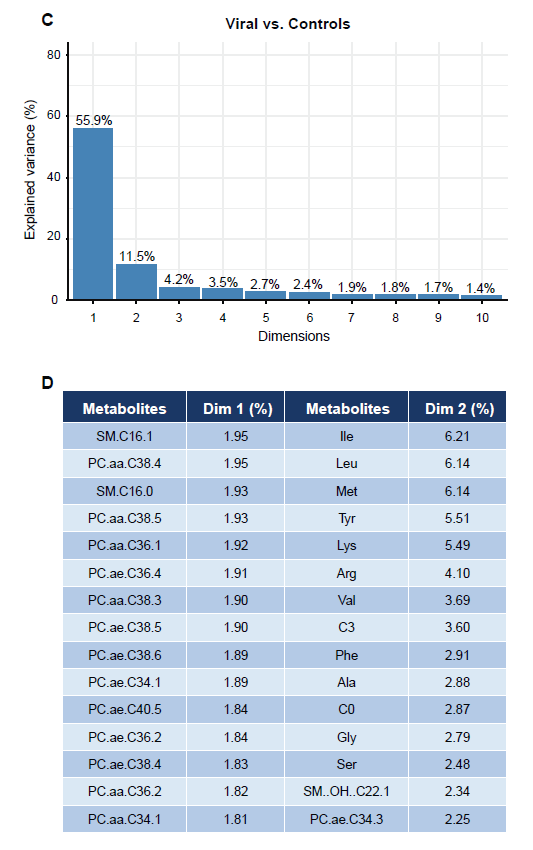


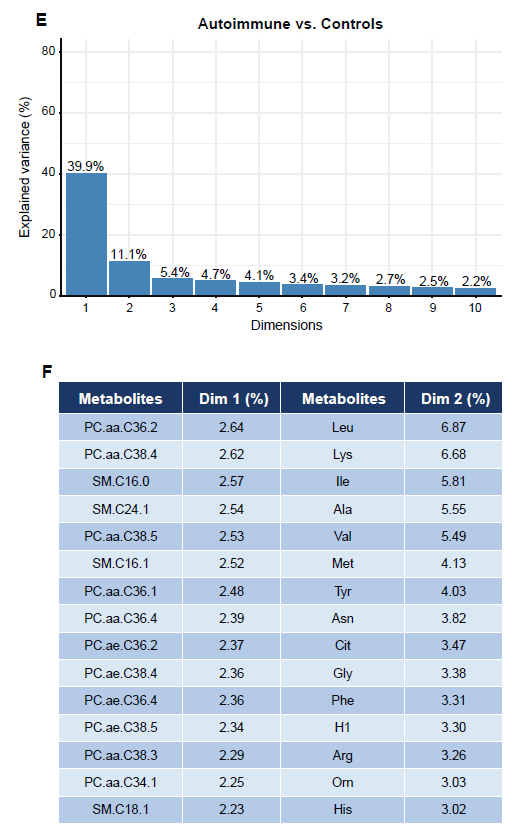


**Fig. S3. Correlations among concentrations of the 85 CSF metabolites across all samples.** Correlations among all analytes were determined using Pearson correlation analysis. The resulting Pearson correlation coefficients (*ρ)* were then used as input into a hierarchical clustering analysis. The correlation coefficient values are indicated by the color scheme shown in the legend. The arrows point to short-chain acylcarnitines C4 and C5.


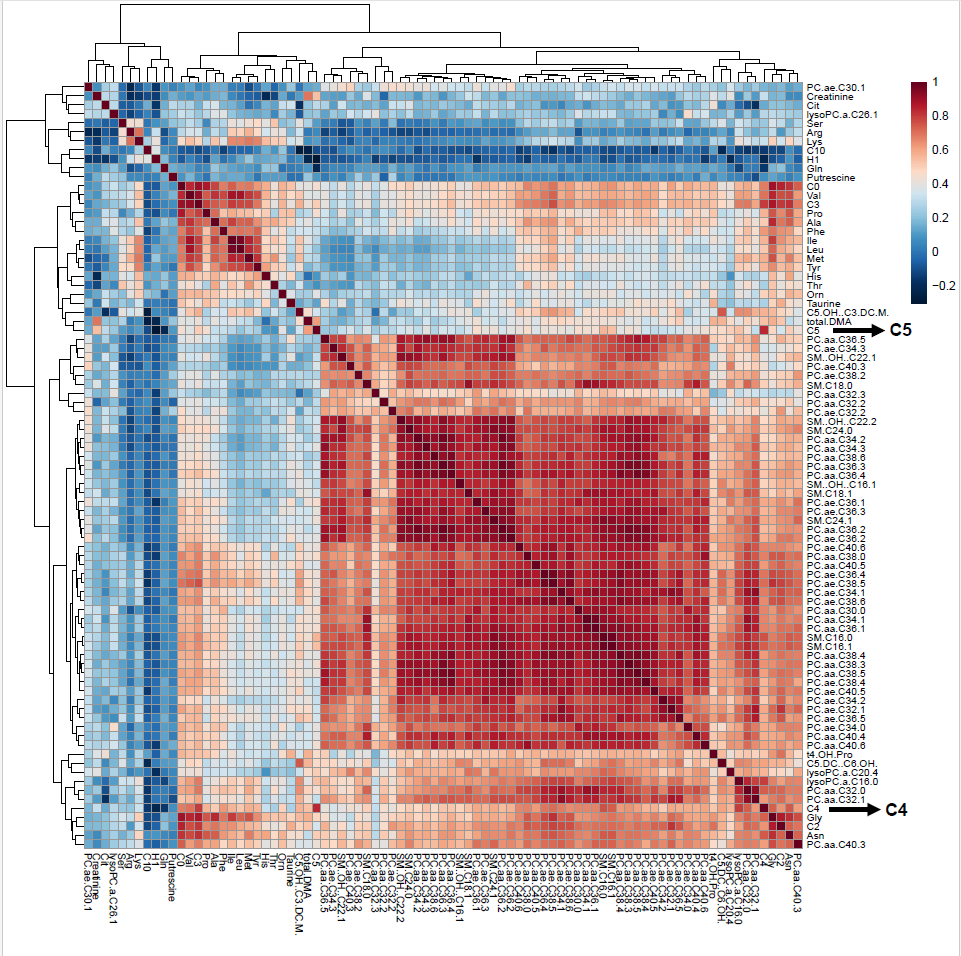
**Fig. S4. HAUCA curve analysis to assess the likelihood of false positive biomarker identification.** The number of biomarkers exceeding a given AUC value in ROC analysis is plotted on the X-axis. The analysis compares the number of biomarkers identified in the real data set (blue curve) to those identified in a random data set of the same variance (black curve), indicating the number of biomarkers expected by chance alone. The red curve delineates the upper bound 95% CI of the random data set. An AUC of 0.8 was used as AUC cut-off to define accurate biomarkers in the ROC curve analysis shown in Fig. 3. **A.** Viral CNS infections vs. autoimmune neuroinflammation. No markers in the random data set are expected to exceed AUC = 0.8, but 38 did so in the real data set. **B**. Viral CNS infections vs. controls. No markers in the random data set are expected to exceed AUC = 0.8, but 37 did so in the real data set. **C.** Autoimmune neuroinflammation vs. controls. No markers in the random or the real data set exceed AUC = 0.8.


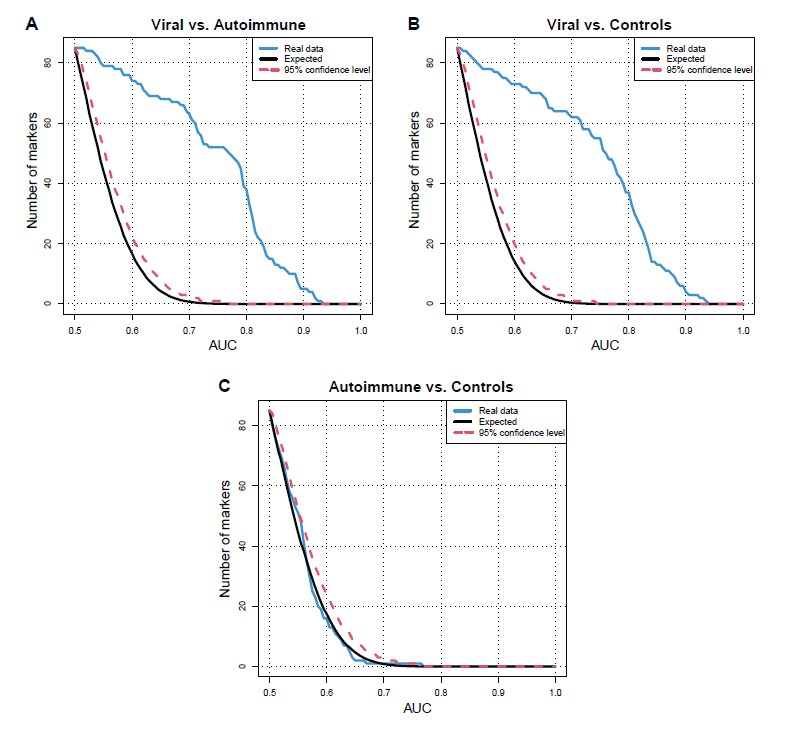


| **Table S1.** Diagnostic criteria and clinical information. | | | |
| --- | --- | --- | --- |
| **Diagnosis** | **Criteria** | **Disease grade/activity** | **Systemic treatment at time of lumbar puncture ^a^** |
| HSV encephalitis (n=9) | Mental status changes and positive HSV PCR or elevated (>1.5) ASI | Encephalitis, 100% | Acyclovir (n=4)  – with ampicillin (n=1) |
| VZV meningitis/encephalitis (n=15) | Detection of VZV in CSF by PCR and/or intrathecal synthesis of VZV IgG,  clinical meningitis/encephalitis with or without typical zoster rash | Meningitis, 73%  Encephalitis, 27% | Immunosuppression (n=1)  – rituximab, bendamustin (3weeks prior to lumbar puncture due to mantle cell lymphoma) |
| Enterovirus meningitis | Clinical meningitis and detection of enterovirus in CSF by PCR | Acute onset (100%)  (symptoms ≤3 months) | — |
| Anti-NMDA-R encephalitis (n=8) | Clinical encephalitis and detection of IgG anti-NMDA-R antibodies in CSF | Acute onset (100%)  (symptoms ≤3 months) | — |
| Multiple sclerosis (n=17)  – RRMS (n=15)  – SPMS (n=2) | McDonald 2017 criteria ^b^ | Acute flare (82%)  Stable (12%) | Corticosteroids (n=2) |
| Tourette syndrome | Criteria according to DSM-5 ^c^ | Classified by YGTSS-TTS  mild (10%)  moderate (70%)  severe (20%) | Symptomatic treatment (n=3)  – Abilify (n=1)  – Dronabinol (n=1)  – Sativex (n=1) |
| Bell’s palsy | Facial nerve palsy without evidence of infectious etiology or CSF pleocytosis | ­— | — |
| ^a^ Excluding antipyretics, analgesics and medications for unrelated conditions  ^b^ Thompson, A et al., Diagnosis of multiple sclerosis: 2017 revisions of the McDonald Criteria. Lancet Neurol 2018; 17(2): 162–173.)  ^c^ American Psychiatric Association (2013) Diagnostic and statistical manual of mental disorders (5th ed.)  Abbreviations: RRMS = relapsing-remitting multiple sclerosis; SPMS= secondary-progressive multiple sclerosis; YGTSS-TTS = Yale Global Tic Severity Scale Total Tic Score. | | | |

| **Table S2.** Demographic and clinical laboratory characteristics of the seven subgroups. | | | | | | | | | |
| --- | --- | --- | --- | --- | --- | --- | --- | --- | --- |
| **Parameter** | | HSV encephalitis  (n=9) | VZV  meningitis / encephalitis  (n=15) | Enterovirus  meningitis  (n=10) | Multiple sclerosis  (n=17) | NMDA  (n=8) | Tourette syndrome  (n=20) | Bell’s palsy  (n=11) |  |
|  |  |  | **Median** (range) | | | | | | ***P* value** |
| Age (years) | | 56 (29 – 76) | 51 (13 – 80) | 39 (22 – 76) | 33  (23-58) | 27  (19-69) | 38.5  (19-64) | 45  (22-83) | 0.05^a^ |
| Sex (female %) | | 22 | 35 | 50 | 47 | 85 | 10 | 55 | 0.02^b^ |
| **Blood** | Leukocyte count (1000/µL) | 10.1  (1.8 - 12.8) | 6.9  (3.8 - 13.6) | 7  (4 – 14) | 6.5  (2.8-18.2) | 7  (2.1-9.7) | 6.2  (4.7-8.7) | 8.3  (4.6-11.9) | 0.5^a^ |
|  | C-reactive protein (mg/L) | 3 (1 - 102) | 2 (1 - 25) | 4 (1 – 39) | 1 (1-23) | 14.2 (1-37) | ND | 3 (1-31) | 0.04^a^ |
| **CSF** | Cell count (1/µl) | 90.7  (16 - 723) | 60.5  (1.7 - 1536) | 9.2  (0.7 - 619) | 7.7  (0.7-48) | 20  (1.7-172) | 1  (0.3-4.7) | 2  (0.3-4.7) | 6.98e-14^a^ |
|  | Lactate (mmol/L) | 2.3  (1.8 - 3.4) | 2.7  (1.5 - 5.5) | 1.8  (1.6 - 3.6) | 1.6  (1.3-2.4) | 2.2  (1.4-2.6) | 1.6  (1.4-1.9) | 1.6  (1.2-2.2) | 1.5e-06^a^ |
|  | Protein (mg/L) | 1.2  (0.5 - 2.3) | 0.67  (0.42 - 2.1) | 0.5  (0.24 - 0.98) | 0.5  (0.3-0.7) | 0.4  (0.2-0.9) | 0.35  (0.2-0.6) | 0.5  (0.3-0.8) | 6.0e-06^a^ |
|  | Q IgG | 19.6  (4.5 - 58.4) | 9.5  (3.2 - 21.1) | 5  (1.3 - 13.6) | 4.8  (1.6-13.2) | 5.9  (2.2-11.2) | 2.3  (1.3-5) | 2.8  (1.5-6.5) | 1.4e-08^a^ |
|  | IgG Index | 0.76  (0.5 - 1.3) | 0.56  (0.5 - 1.2) | 0.51  (0.47 - 0 .63) | 0.8  (0.6-2.9) | 0.7  (0.5-2.4) | 0.5  (0.5-1) | 0.5  (0.4-0.6) | 9.1e-11^a^ |
|  | Q albumin | 31.8  (6.8 - 44.4) | 12.4  (6.3 - 41.1) | 8.9  (2.7 - 21.7) | 6.2  (2.5-9.6) | 4.4  (2.7-12.3) | 4.7  (2.4-8.6) | 5.5  (3.4-14) | 1.1e-07^a^ |
|  | BCB disruption % | | | | | | | | |
|  | None (1) | 11 | 21 | 25 | 53 | 75 | 80 | 55 | 0.0005^b^ |
|  | Light (2) | 11 | 36 | 62.5 | 47 | 25 | 20 | 45 |  |
|  | Moderate (3) | 11 | 29 | 12.5 | 0 | 0 | 0 | 0 |  |
|  | Severe (4) | 67 | 14 | 0 | 0 | 0 | 0 | 0 |  |
| ^a^ Kruskal - Wallis test ^b^ Chi – squared test. HSV = herpes simplex virus; NMDA = anti-NMDA-receptor autoimmune encephalitis; ND = not determined; VZV = varicella zoster virus. | | | | | | | | | |

| **Table S3:** Differences among the metabolite classes in biomarker potential.**^a^** | | | | |
| --- | --- | --- | --- | --- |
|  | | | | |
| **Viral CNS infection vs. autoimmune neuroinflammation** | | | | |
| **Metabolite class** | **No. included in analysis** | **No. qualifying as accurate biomarker^b^** | **% of all analytes** | **% of metabolite class** |
| All | 85 | 38 | 45 | n/a |
| Sphingomyelins | 9 | 8 | 9.4 | 89 |
| Acylcarnitines | 8 | 5 | 5.9 | 63 |
| Phosphatidylcholines | 41 | 21 | 25 | 51 |
| Lysophosphatidylcholines | 3 | 1 | 1.2 | 33 |
| Amino acids | 18 | 3 | 3.5 | 17 |
| Amino acid metabolites | 5 | 0 | 0 | 0 |
|  | | | | |
| **Viral CNS infection vs. controls** | | | | |
| **Metabolite class** | **No. included in analysis** | **No. qualifying as accurate biomarker^b^** | **% of all analytes** | **% of metabolite class** |
| All | 85 | 37 | 44 | n/a |
| Sphingomyelins | 9 | 7 | 8 | 78 |
| Phosphatidylcholines | 8 | 5 | 6 | 63 |
| Acylcarnitines | 41 | 22 | 26 | 54 |
| Amino acids | 18 | 3 | 3.5 | 17 |
| Amino acid metabolites | 5 | 0 | 0 | 0 |
| Lysophosphatidylcholines | 3 | 0 | 0 | 0 |
|  | | | | |
| **Autoimmune neuroinflammation vs. controls** | | | | |
| **Metabolite class** | **No. included in analysis** | **No. qualifying as biomarker^b^** | **% of all analytes** | **% of metabolite class** |
| All | 85 | 0 | 0 | 0 |
| Phosphatidylcholines | 41 | 0 | 0 | 0 |
| Amino acids | 18 | 0 | 0 | 0 |
| Sphingomyelins | 9 | 0 | 0 | 0 |
| Acylcarnitines | 8 | 0 | 0 | 0 |
| Amino acid metabolites | 5 | 0 | 0 | 0 |
| Lysophosphatidylcholines | 3 | 0 | 0 | 0 |
| ^a^ Analysis based on the ROC analysis presented in Fig 2. The metabolite classes are ranked according to “% of class” (indicating biomarker potential within this class) in descending order.  ^b^ AUC ≥ 0.8, lower bound AUC 95% CI ≥ 0.5, and p < 0.05 for asymptotic significance of ROC curve. | | | | |

|  |  | | | | | | |
| --- | --- | --- | --- | --- | --- | --- | --- |
| **Table S4.** Comparison of standard parameters and the 10 most robust CSF metabolite biomarkers to differentiate between viral CNS infections and controls | | | | | | | |
| **Standard parameters** | | | | **CSF Metabolites** | | | |
| **Parameter** | | **AUC**  **(95% CI)** | **Ratio of means** | **Metabolite** | **AUC**  **(95% CI)** | **Ratio of means** | **Selection frequency^a^** |
| CSF cell count | | 0.93***  (0.86-0.98) | 104.6 | SM.C16.0 | 0.94***  (0.88-0.99) | 3.1 | 1.0 |
| Q IgG | | 0.9***  (0.83-0.97) | 4.1 | PC.aa.C30.0 | 0.93***  (0.87-0.98) | 2.1 | 1.0 |
| Q albumin | | 0.9***  (0.82-0.96) | 3.0 | C5 | 0.92***  (0.85-0.97) | 5.3 | 1.0 |
| CSF lactate | | 0.85***  (0.75-0.94) | 1.5 | SM.C16.1 | 0.90***  (0.82-0.97) | 2.8 | 1.0 |
| CSF protein | | 0.81***  (0.71-0.92) | 2.1 | Gly | 0.90***  (0.8-0.97) | 3.2 | 1.0 |
| BCB disruption | | 0.81***  (0.72-0.9) | n/a | PC.ae.C34.0 | 0.90***  (0.81-0.96) | 2.3 | 1.0 |
| IgG-index | | 0.75***  (0.62-0.88) | 1.2 | PC.aa.C38.0 | 0.89***  (0.80-0.95) | 2.4 | 0.98 |
| Blood  CRP | | 0.56  (0.38-0.75) | 2.6 | C4 | 0.88***  (0.79-0.95) | 3.4 | 0.98 |
| Blood  leukocytes | | 0.55  (0.41-0.69) | 1.1 | PC.ae.C36.2 | 0.88***  (0.78-0.95) | 2.5 | 0.98 |
|  | |  |  | PC.aa.C34.3 | 0.88***  (0.79-0.95) | 3.4 | 0.86 |
| ^a^ Frequency of selection among top 10 biomarkers in leave-one-out (jackknife) cross-validation. 1 = always selected, 0 = never selected. ** *p* ≤0.01; *** *p* ≤0.001. | | | | | | | |

|  |  | | | | | | |
| --- | --- | --- | --- | --- | --- | --- | --- |
| **Table S5.** Comparison of standard parameters and the 10 most robust CSF metabolite biomarkers to differentiate between autoimmune neuroinflammation and controls. | | | | | | | |
| **Standard parameters** | | | | **CSF Metabolites** | | | |
| **Parameter** | | **AUC**  **(95% CI)** | **Ratio of means** | **Metabolite** | **AUC**  **(95% CI)** | **Ratio of means** | **Selection frequency^a^** |
| IgG index | | 0.90***  (0.81-0.92) | 1.9 | Pro | 0.77***  (0.63-0.88) | 1.7 | 1.0 |
| CSF cell count | | 0.89***  (0.80-0.97) | 12 | Total DMA | 0.67*  (0.52-0.80) | 1.2 | 1.0 |
| Q IgG | | 0.83***  (0.71-0.92) | 2.2 | C5.OH..C3.DC.M. | 0.65  (0.52-0.78) | 1.1 | 1.0 |
| CSF lactate | | 0.60  (0.43-0.77) | 1.1 | PC.ae.C34.1 | 0.64  (0.48-0.8) | 1.2 | 0.98 |
| Q albumin | | 0.60  (0.45-0.75) | 1.1 | Tyr | 0.64  (0.48-0.79) | 0.86 | 0.98 |
| CSF  protein | | 0.59  (0.44-0.73) | 1.1 | PC.aa.C40.6 | 0.64  (0.52-0.76) | 0.89 | 1.0 |
| BCB disruption | | 0.46  (0.32-0.68) | – | PC.ae.C32.2 | 0.64  (0.51-0.75) | 0.77 | 1.0 |
| Blood CRP | | 0.50  (0.30-0.73) | 1.2 | Gly | 0.63  (0.46-0.8) | 1.2 | 0.80 |
| Blood  leukocytes | | 0.49  (0.33-0.69) | 1.1 | C10 | 0.63  (0.48-0.77) | 0.85 | 0.66 |
|  | |  |  | PC.aa.C32.1 | 0.62  (0.46-0.77) | 1. 2 | 0.41 |
| ^a^ Frequency of selection among top 10 biomarkers in leave-one-out (jackknife) cross-validation. 1 = always selected, 0 = never selected. * *p* ≤0.05; ** *p* ≤0.01; *** *p* ≤0.001. | | | | | | | |

| **Table S6.** Comparison of common blood and CSF standard diagnostic parameters and the top five CSF metabolite biomarkers: sensitivity, specificity, PPV, and NPV for viral CNS infections vs. controls.^a^ | | | | | | | |
| --- | --- | --- | --- | --- | --- | --- | --- |
| **Biomarker** | | **Sensitivity** | **Specificity** | **PPV** | **NPV** | **Cut-off value** | **Youden index^a^** |
| Blood | Leukocytes | 0.35 | 0.90 | 0.8 | 0.56 | 9.8 (1000/µL) | 0.25 |
|  | CRP | 0.29 | 0.91 | 0.91 | 0.29 | 9 mg/L | 0.20 |
| CSF standard parameters | Cell count | 0.82 | 1 | 1 | 0.84 | 5 cells/µl | 0.82 |
|  | Q IgG | 0.88 | 0.81 | 0.83 | 0.86 | 4.03 | 0.69 |
|  | Qalb | 0.97 | 0.71 | 0.79 | 0.96 | 6.3 | 0.68 |
|  | CSF lactate | 0.79 | 0.84 | 0.84 | 0.79 | 1.8 mmol/L | 0.63 |
|  | Protein | 0.88 | 0.68 | 0.75 | 0.84 | 0.46 mg/L | 0.56 |
|  | BCB disruption | 0.79 | 0.71 | 0.75 | 0.76 | 2 | 0.50 |
|  | IgG Index | 0.61 | 0.81 | 0.78 | 0.66 | 0.54 | 0.42 |
| CSF metabolites | SM.C16.0 | 0.82 | 0.94 | 0.93 | 0.83 | 0.5 µM | 0.76 |
|  | SM.C16.1 | 0.76 | 0.97 | 0.96 | 0.79 | 0.06 µM | 0.73 |
|  | Gly | 0.76 | 0.97 | 0.96 | 0.79 | 8.4 µM | 0.73 |
|  | PC.aa.C30.0 | 0.94 | 0.77 | 0.82 | 0.92 | 0.06 µM | 0.71 |
|  | C5 | 0.91 | 0.77 | 0.82 | 0.89 | 0.02 µM | 0.68 |
| ^a^ Within each class (blood parameters, standard CSF parameters, CSF metabolites), markers are ranked by Youden index (sensitivity + specificity - 1 at the optimal cut-off point in the ROC curve) in descending order. | | | | | | | |

| **Table S7.** Comparison of common blood and CSF standard diagnostic parameters and the top five CSF metabolite biomarkers: sensitivity, specificity, PPV, and NPV for autoimmune neuroinflammation vs. controls.^a^ | | | | | | | |
| --- | --- | --- | --- | --- | --- | --- | --- |
| **Biomarker** | | **Sensitivity** | **Specificity** | **PPV** | **NPV** | **Cut-off value** | **Youden index^a^** |
| Blood | Leukocytes | 0.32 | 0.9 | 0.73 | 0.62 | 8.8 (1000/µL) | 0.22 |
|  | CRP | 0.28 | 0.91 | 0.88 | 0.36 | 8.1 mg/L | 0.19 |
| CSF standard parameters | IgG Index | 0.88 | 0.84 | 0.81 | 0.9 | 0.55 | 0.72 |
|  | Cell count | 0.68 | 1 | 1 | 0.79 | 5.3 cells/µl | 0.68 |
|  | Q IgG | 0.8 | 0.81 | 0.77 | 0.83 | 3.7 | 0.61 |
|  | CSF lactate | 0.36 | 0.97 | 0.9 | 0.65 | 2.1 mmol/L | 0.33 |
|  | Qalb | 0.64 | 0.65 | 0.59 | 0.69 | 5.6 | 0.29 |
|  | CSF protein | 0.6 | 0.61 | 0.56 | 0.66 | 0.4 mg/L | 0.21 |
|  | BCB disruption | 0.4 | 0.71 | 0.53 | 0.59 | 2 | 0.11 |
| CSF metabolites | Pro | 0.76 | 0.68 | 0.66 | 0.78 | 1.2 µM | 0.44 |
|  | PC.ae.C34.1 | 0.76 | 0.61 | 0.61 | 0.76 | 0.06 µM | 0.37 |
|  | Total DMA | 0.44 | 0.90 | 0.79 | 0.67 | 0.24 µM | 0.34 |
|  | C5.OH..C3.DC.M. | 0.72 | 0.52 | 0.55 | 0.70 | 0.02 µM | 0.24 |
|  | Tyr | 0.24 | 0.81 | 0.5 | 0.57 | 12.9 µM | 0.05 |
| ^a^ Within each class (blood parameters, standard CSF parameters, CSF metabolites), markers are ranked by Youden index (sensitivity + specificity - 1 at the optimal cut-off point in the ROC curve) in descending order. | | | | | | | |

| **Table S8.** Sex-specific concentrations in Bell’s palsy CSF samples of the top 6 CSF metabolite biomarkers for the differentiation between viral CNS infections and autoimmune inflammation. | | |
| --- | --- | --- |
| **Metabolite** | **Ratio female /male** | ***P* value ^a^** |
| C4 | 0.77 | 0.41 |
| C5 | 0.81 | 0.23 |
| PC.aa.C30.0 | 1.30 | 0.02 |
| PC.ae.C32.2 | 0.95 | 0.86 |
| PC.aa.C38.0 | 1.09 | 0.76 |
| SM.C16.0 | 0.79 | 0.03 |
| ^a^ Uncorrected *p* values, unpaired T-test.  Female n = 6, male n= 5. | | |
